# Multielemental Stoichiometry in Plant Organs: A Case Study With the Alpine Herb *Gentiana rigescens* Across Southwest China

**DOI:** 10.3389/fpls.2020.00441

**Published:** 2020-04-28

**Authors:** Ji Zhang, Yuanzhong Wang, Chuantao Cai

**Affiliations:** ^1^Chinese Academy of Sciences (CAS) Key Laboratory of Tropical Plant Resources and Sustainable Use, Xishuangbanna Tropical Botanical Garden, Chinese Academy of Sciences, Menglun, China; ^2^Center of Economic Botany, Core Botanical Gardens, Chinese Academy of Sciences, Menglun, China; ^3^College of Life Sciences, University of Chinese Academy of Sciences, Beijing, China; ^4^Medicinal Plants Research Institute, Yunnan Academy of Agricultural Sciences, Kunming, China

**Keywords:** homeostasis, nutrient, herbaceous plant, reproduction, soil, climate

## Abstract

Multiple elements are required to be allocated to different organs to meet the demands for plant growth, reproduction, and maintenance. However, our knowledge remains limited on the stoichiometry in all plant organs in response to heterogeneous environments. Here, we present the systematic investigation of multielemental stoichiometry in organs of the alpine plant *Gentiana rigescens* across different environmental conditions. The slopes of N–P stoichiometric relationships among organs in *G. rigescens* did not differ significantly between environments even in flowers, the most active organ with the highest N and P level. C:P ratios had strong positive relationships with N:P ratios within and between organs. Zn had strong positive correlations with Fe, S, or Cu in each organ, indicating the potential interactions among the homeostases of these elements. The contents of macroelements, such as C, N, P, Ca, Mg, and S, were higher in plant organs than those in soil and exhibited a relatively narrow range in plant organs. However, *G. rigescens* reduced Fe uptake from soil and showed the strictest homeostasis in its root, implying its resistance to excess Fe. Furthermore, precipitation and temperature associated with geography, followed by soil P, were the main divers for the multielemental stoichiometry in this species. Plant stoichiometry responded differently to abiotic environmental factors, depending on organ type and element. N:P ratio, no matter in which organ, showed little flexibility to climate factors. The results have implications for understanding the regulation of multielemental stoichiometry in plant individuals to environmental changes. Further studies are needed on the interactions of multielement homeostasis in plants.

## Introduction

Ecological stoichiometry offers a framework to understand the balance of multiple elements in ecological interactions and processes (Elser et al., 2010). Under this framework, the degree to which organisms maintain a constant elemental composition in response to the availability of their environmental resources is referred to as “stoichiometric homeostasis” (Sterner and Elser, 2002). For example, an organism’s carbon/nitrogeN:Phosphorus stoichiometry tends to be more constrained than the stoichiometry of its environment (Sardans et al., 2012). In addition, the core central tendency in N:P ratio is similar among observations in different biota, which could be due to the similar biochemical investment across all biota (Zhang and Elser, 2017). Apart from C, N, and P, a more complete understanding of homeostasis necessitates the consideration of other elements, especially the nutrient elements for plant growth (Jeyasingh et al., 2017; Ågren and Weih, 2020). Potassium (K) is the second most abundant nutrient in plant photosynthetic tissues and is required for many functions, such as the maintenance of electrical potentials across cell membranes (Britto and Kronzucker, 2008; Sardans and Peñuelas, 2015). Calcium (Ca) is another essential nutrient for plants and is required for structural roles in the membranes and cell wall (White and Broadley, 2003). Magnesium (Mg) is vital for the function of many cellular enzymes and for the aggregation of ribosomes, and the maintenance of its homeostasis in the plant is essential for viability (Shaul, 2002). Sulfur (S) plays critical roles in many biological processes, including the role linked to trace element homeostasis in plants (Na and Salt, 2011). Iron (Fe) is an essential cofactor for fundamental biochemical activities, so Fe homeostasis plays an important role in plant nutrition (Vigani et al., 2013). Plants only need small amounts of manganese (Mn), which vary more than two orders of magnitude in soils (Shao et al., 2017); therefore, plants must maintain their Mn homeostasis for health growth. Copper (Cu) is one of the essential trace elements for plants and is a cofactor in proteins that are involved in electron transfer reactions (Burkhead et al., 2009). Zinc (Zn) with multifunctions in all plants has key roles in basal metabolism, defense, and virulence (Cabot et al., 2019).

When nutrients are taken up by plants, plentiful processes regulate elemental homeostasis to maintain plant function (Sperfeld et al., 2017). Multiple elements are required to be allocated to different organs to meet the demands for plant growth, reproduction, and maintenance (Wang et al., 2012). It is necessary to consider all plant organs in understanding how plants respond to nutrient limitation and changing environmental conditions (Allen and Gillooly, 2009; Kleyer and Minden, 2015). In a previous study, distinct patterns of C:N:P stoichiometry have been found in plant organs related to their internal function (Minden and Kleyer, 2014). Stoichiometric homeostasis for N and P has been found among vegetative tissues in forest plant (Zhang et al., 2018a). However, relatively little is known about multielemental stoichiometry at organ level, especially in reproductive tissue, in whole plant individuals.

Many plant species are distributed at relatively wide spatial scales and are, therefore, exposed to heterogeneous environments (Helsen et al., 2017). An understanding of stoichiometry within individuals is a key component for the accurate prediction of responses of plants to environmental change (Sistla and Schimel, 2012). Soil nutrient availability, as well as plant nutrient demand under climate change, can affect leaf element contents and their coupling (Tian et al., 2019). Several systematic investigations or meta-analysis studies have been done on the effects of soil and climate on plant C:N:P stoichiometry at global scale (Reich and Oleksyn, 2004; Sardans et al., 2017), regional scale (Yang et al., 2015; Zhang et al., 2018b), or local scale (Zhao et al., 2014). Some studies on plant stoichiometry that go beyond C, N, and P under different environmental conditions were also carried out. For example, Han et al. (2011) found that variation in leaf elemental content was more constrained for nutrients with the highest requirements. In a fertilizer experiment with different *Salix* spp. genotypes, difference in the elemental contents in the environment was the major driver of variation in leaf nutrient relations (Ågren and Weih, 2012). However, our knowledge remains limited on multielemental stoichiometry in plant organs to nutrient stress or climate change.

To address these gaps in our knowledge, here, we present the systematic investigation of multielemental stoichiometry in organs of an alpine plant across different environmental conditions. Our questions are as follows. Does multielemental stoichiometry differ among organs? How does the plant regulate the elemental stoichiometry to response soil nutrient heterogeneity? Is multielemental stoichiometry in plants affected by different climates? Answering these questions will help us understand how plant regulates its multielemental stoichiometry in response to heterogeneous environments.

## Materials and Methods

**FIGURE 1:**
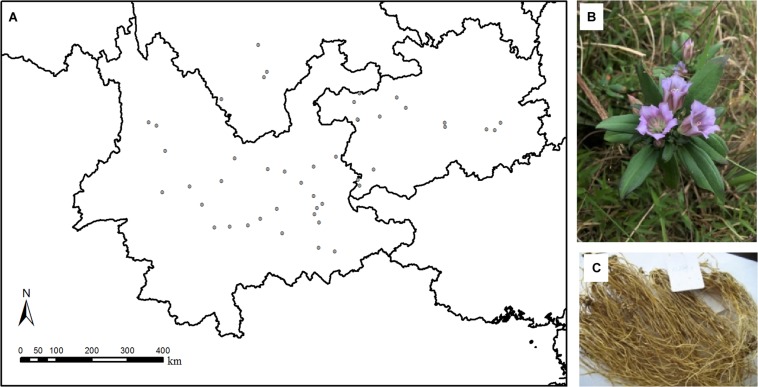
Sampling sites and samples of *Gentiana rigescens*. **(A)** Sampling sites of the plant species in southwest of China. **(B)** Above-ground part of the plant species. **(C)** Below-ground part of the plant species.

Our investigations were conducted in Yunnan–Guizhou Plateau, southwest China. This area is very close to the Qinghai–Tibet Plateau, which is considered to be a geographical source area for genus *Gentiana* (Favre et al., 2016). *Gentiana rigescens* Franch. ex Hemsl. (Gentianaceae) is a perennial plant distributed in the elevation range of 1,100–3,000 m in subtropical evergreen broad-leaved and sclerophyllous forests, subtropical conifer forest, and subtropical mixed deciduous-evergreen shrubland in southwest China (Li and Walker, 1986; Flora of China Editorial Committee, 1988). From December 2012 to January 2013 and October to November 2013 (within the populations’ flowering period), we investigated 45 wild populations of *G. rigescens* in Yunnan, Guizhou, and Sichuan provinces, China in the elevation range of 1,230–2,980 m and in the latitude range of 23.316–28.524°N (Figure 1 and Supplementary Table S1). In each sampling site, 10 plant individuals at the flowering stage were dug out carefully with roots from soil and washed. Each individual was divided into roots, stems, leaves, and flowers. The same plant organs from the same sampling site were pooled, which means that there were totally 45 samples for each plant organ. The plant samples were dried to constant mass at 60°C, powdered, screened (<0.125 mm), and kept in polyethylene bag for chemical analyses. From each sampling site, five subsamples of top soil (0–20 cm) were bulked into one soil sample, except two sites. Therefore, totally, 43 soil samples across southwest China were collected. After removing the debris, the samples were air dried and screened (<0.125 mm). The mean annual temperature (MAT) and the mean annual precipitation (MAP) data of the sampling sites in 2012 were obtained from a temperature and precipitation interpolated 1 km spatial resolution raster data (Wang et al., 2017).

The soil and plant samples were kept at 60°C for 24 h using an electrically heated laboratory oven before analyses. Total C and N contents in the samples were directly determined by an elemental analyzer (Model Vario MAX CN, Elementar Analysensysteme GmbH, Germany). Total contents of P, K, Ca, Mg, S, Fe, Mn, Cur, and Zn were determined by an inductively coupled plasma atomic-emission spectrometer (Model iCAP6300, Thermo Fisher Scientific, United States) after wet digestion. Several standard reference materials, such as GBW 10015 (GSB-6) spinach and GBW 10052 (GSB-30) green tea, were purchased from the National Research Center for Certified Reference Materials of China to verify the elemental analysis methods. A digital pH meter (Model FE20, Mettler-Toledo International Inc., China) was used to determine the soil pH. Soil organic matter content was determined by the potassium dichromate volumetric method.

Raw data are available in Supplementary Data Sheet S1. Data were log10 transformed before statistical analysis. Linear regression models of the form

log(y)=alog(x)+logb

were used for the stoichiometric relationships defined as log*y* and log*x*, where *x* is the P content (or element contents in soils), *y* is the N content (or element contents in plants), *a* is the slope, and log*b* is the intercept. The strength of elemental homeostasis is expressed as the homeostasis regulation coefficient *H*, which is calculated as 1/slope (Sterner and Elser, 2002). We estimated the stoichiometric relationships by standardized major axis regression and tested for a common slope among several stoichiometric relations using R software version 3.5.3 package “smatr” version 3.4-8 (Warton et al., 2012; R Core Team, 2019). A heatmap integrated with dendrogram was performed to explore correlations of the element contents and C, N, and P ratios in soils and intraorgan using R package “pheatmap” version 1.0.12. Multiple factor analysis was employed to investigate different groups (climate, coordinate, and soil) of abiotic environmental factors on plant multielemental stoichiometry using R packages “FactoMineR” version 1.34 and “factoextra” version 1.0.5. One-way ANOVA with Tukey *post hoc* test was performed to compare element contents among different samples.

## Results

In the soils, the mean content was 3.4% for C, 0.19% for N, and 0.056% for P; in the plants, the mean content varied from 45.2 to 46.9% for C, 0.080 to 1.44% for N, and 0.085 to 0.189% for P (Table 1). N contents were significantly (*p* < 0.05) higher in flower and leaf than in stem and root, while P content in flower was significantly (*p* < 0.05) higher than the values in other plant organs (Table 1).

**TABLE 1:** Contents and molar ratios of C, N, and P in soils and plants.

	*n*	%C	%N	%P	C:N:P
Soil	43	3.4 ± 2.4b	0.19 ± 0.13c	0.056 ± 0.043d	169:8:1
Root	45	44.4 ± 1.5a	0.89 ± 0.21b	0.100 ± 0.047bc	1,390:23:1
Stem	45	46.2 ± 0.7a	0.80 ± 0.18b	0.085 ± 0.046c	1,746:25:1
Leaf	45	45.2 ± 1.1a	1.29 ± 0.36a	0.134 ± 0.076b	1,157:26:1
Flower	45	46.9 ± 0.9a	1.44 ± 0.31a	0.189 ± 0.068a	728:18:1

*Lowercase letters in the same columnn indicate significant differences at 0.05 level.*

**FIGURE 2:**
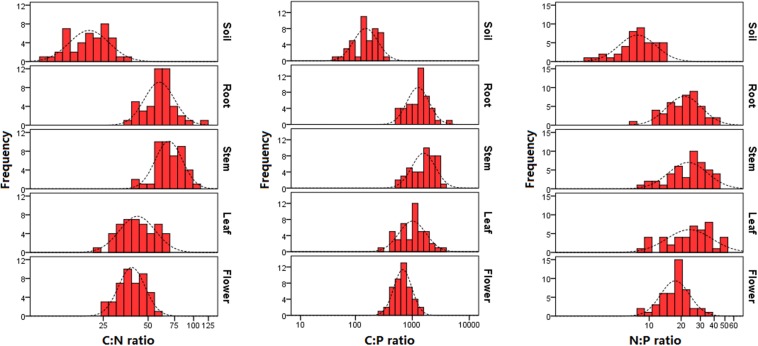
Distribution of C:N, C:P, and N:P molar ratios in soils and plants. The sample size is 43 for soil and 45 for each plant organ.

C:N ratio varied from 10 to 35 in soil, 39 to 114 in root, 39 to 100 in stem, 23 to 69 in leaf, and 24 to 56 in flower; C:P ratio varied from 45 to 334 in soil, 513 to 4,067 in root, 506 to 3,185 in stem, 308 to 3,343 in leaf, and 315 to 1,564 in flower; N:P ratio varied from 2 to 15 (with 8 on average) in soil, 6 to 42 (with 23 on average) in root, 9 to 41 (with 25 on average) in stem, 8 to 53 (with 26 on average) in leaf, and 8 to 34 (with 18 on average) in flower (Table 1 and Figure 2).

**FIGURE 3:**
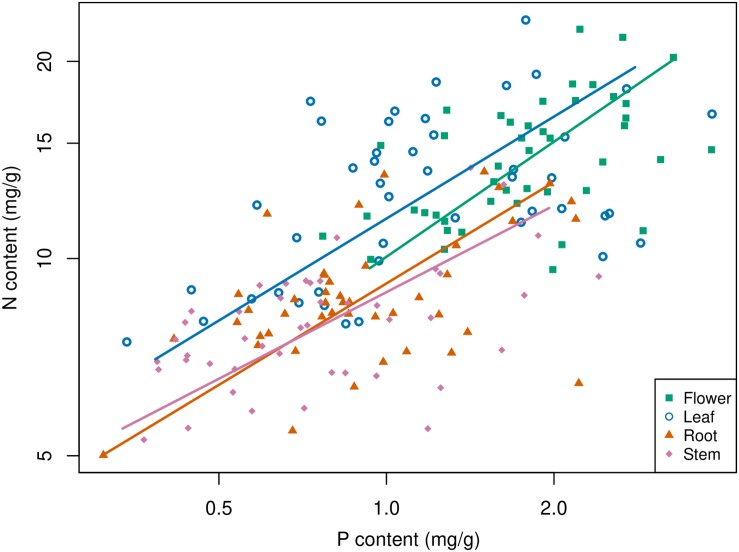
Relationships between N and P in different plant organs. A base-10 logarithmic scale is used for the axes. The sample size is 45 for each plant organ.

**FIGURE 4:**
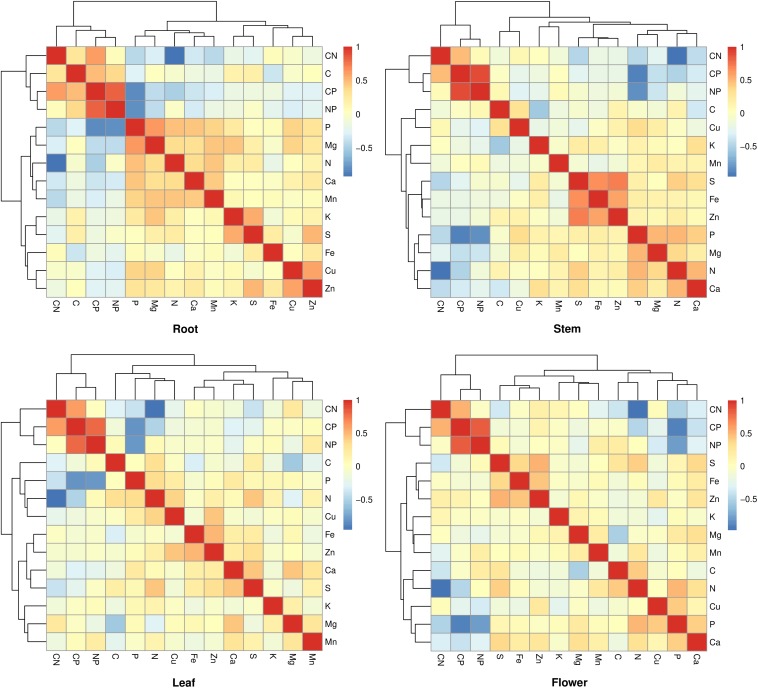
Pearson’s correlation coefficients for the element contents and C, N, and P molar ratios in plant organs. The sample size is 45 for each plant organ.

C:P ratios have strong positive relationships with N:P ratios within and between organs (*p* < 0.01); however, C:N only has strong positive relationship with C:P in root or stem (*p* < 0.01), respectively (Table 2). The largest Pearson’s correlation coefficient, 0.912, was found between leaf N:P ratio and flower N:P ratio, followed by 0.904, which was found between leaf N:P ratio and stem N:P ratio (Table 2). Stoichiometric relationships for N and P contents have been found among different organs, and these relationships shared a common slope (*p* = 0.51) (Figure 3 and Table 3). Zn had strong positive correlations with Fe, S, or Cu in each organ (Figure 4).

**TABLE 2:** Pearson correlation coefficients of C:N, C:P, and N:P ratios in plants.

		Root			Stem			Leaf			Flower		
		C:N	C:P	N:P	C:N	C:P	N:P	C:N	C:P	N:P	C:N	C:P	N:P
Root	C:N	1											
	C:P	0.598*	1										
	N:P	0.071	0.810*	1									
Stem	C:N	0.218	0.077	-0.072	1								
	C:P	0.312	0.703*	0.684*	0.471*	1							
	N:P	0.215	0.779*	0.863*	0.067	0.899*	1						
Leaf	C:N	0.116	0.167	0.070	0.252	0.344	0.238	1					
	C:P	0.311	0.746*	0.723*	0.132	0.776*	0.821*	0.590*	1				
	N:P	0.242	0.798*	0.886*	−0.007	0.764*	0.904*	0.021	0.794*	1			
Flower	C:N	−0.058	0.002	−0.048	0.180	0.190	0.103	0.768*	0.359	−0.102	1		
	C:P	0.216	0.662*	0.674*	0.066	0.683*	0.753*	0.562*	0.898*	0.709*	0.520*	1	
	N:P	0.221	0.763*	0.859*	−0.045	0.694*	0.846*	0.135	0.795*	0.912*	−0.010	0.835*	1

* p < 0.01.

**TABLE 3:** Parameters for the stoichiometric relationships between N and P in plant organs.

	Slope	95% CI of slope	Intercept	95% CI of intercept	*N*	*R*^2^	*p*
Root	0.51	0.40–0.67	0.48	0.33–0.62	45	0.25	<0.001
Stem	0.44	0.34–0.57	0.39	0.25–0.52	45	0.25	<0.001
Leaf	0.52	0.40–0.67	0.58	0.44–0.71	45	0.24	<0.001
Flower	0.58	0.45–0.76	0.59	0.47–0.70	45	0.27	<0.001

**FIGURE 5:**
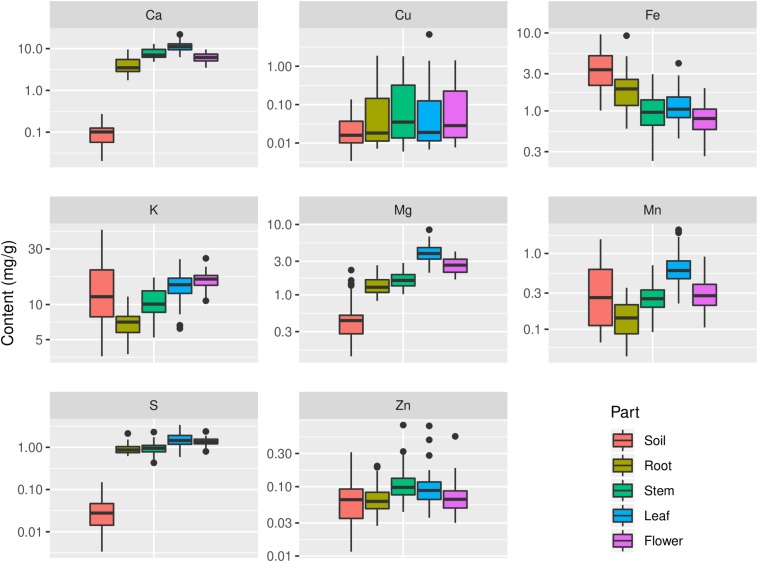
Distribution of contents of Ca, Cu, Fe, K, Mg, Mn, S, and Zn in soils and plants. The sample size is 43 for soil and 45 for each plant organ.

Apparently contents of C, N, P, Ca, Mg, and S in plant organs were significantly higher than their contents in soil (*p* < 0.05), while Fe content in plant organs was significantly lower than its content in soil (*p* < 0.05) (Table 1 and Figure 5). Cu content in plant organs varied more than its content in soil; in contrast, K content varied more in soil samples compared with its contents in plant organs (Figure 5). Mn content in leaf was significantly higher than that in soil, but it was significantly lower in root than that in soil (*p* < 0.05) (Figure 5). Zn content in stem was significantly higher than its content in soil (*p* < 0.05) (Figure 5).

**FIGURE 6:**
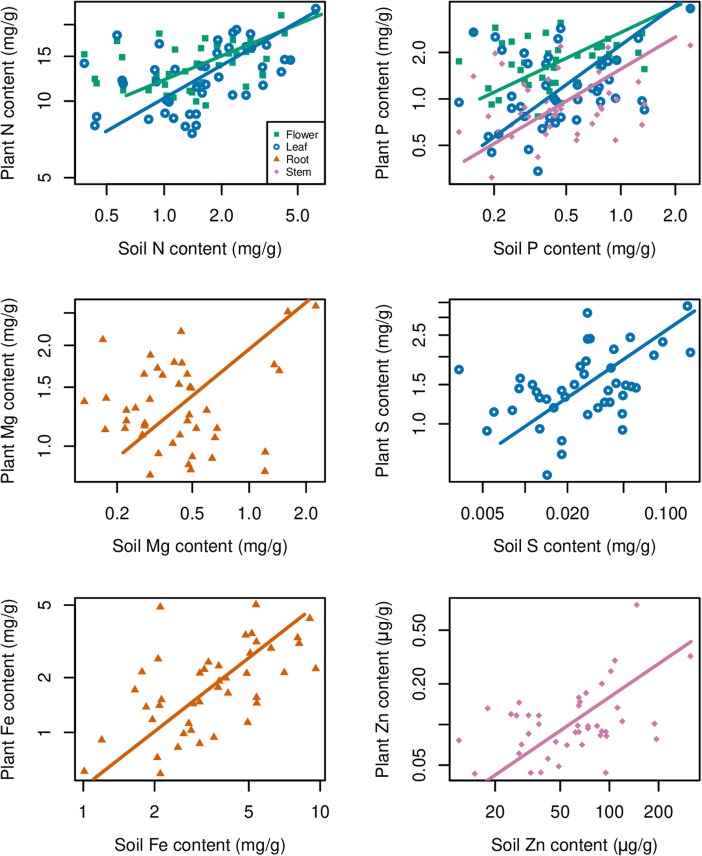
Relationships between soil elements contents and plant elements contents. A base-10 logarithmic scale is used for the axes. The sample size is 43 for both soil and each plant organ, *p* < 0.01.

Contents of some elements in certain organs showed significant relationships with their contents in soil (*p* < 0.01), such as Mg and Fe in root, P and Zn in stem, N, P, and, S in leaf, and N and P in flower (Figure 6). In the above relationships, Fe in root showed the strictest homeostasis (*H* = 1.93), followed by Mg in root (*H* = 1.01), whereas S in leaf showed the weakest homeostasis (*H* = 0.05) (Table 4).

**TABLE 4:** Parameters of relationships between soil elements contents and plant elements contents.

Element	Part	*H*	Slope	95% CI of slope	Intercept	95% CI of intercept	*n*	*R*^2^	*p*
N	Flower	0.41	2.417	1.861–3.141	9.599	8.077–11.120	43	0.30	<0.001
	Leaf	0.34	2.940	2.258–3.827	7.295	5.428–9.162	43	0.28	<0.001
P	Flower	0.65	1.529	1.158–2.018	0.988	0.667–1.158	43	0.21	0.002
	Leaf	0.56	1.793	1.349–2.384	0.311	-0.078–1.349	43	0.16	0.007
	Stem	0.92	1.084	0.819–1.435	0.226	-0.004–0.457	43	0.19	0.004
Mg	Root	1.01	0.987	0.742–1.312	0.846	0.633–1.059	43	0.16	0.007
S	Leaf	0.05	18.505	14.220–24.083	0.898	0.647–1.148	43	0.29	<0.001
Fe	Root	1.93	0.517	0.397–0.675	-0.020	-0.659–0.618	43	0.28	<0.001
Zn	Stem	0.47	2.106	1.591–2.788	-29.906	-89.029–29.217	43	0.19	0.004

**FIGURE 7:**
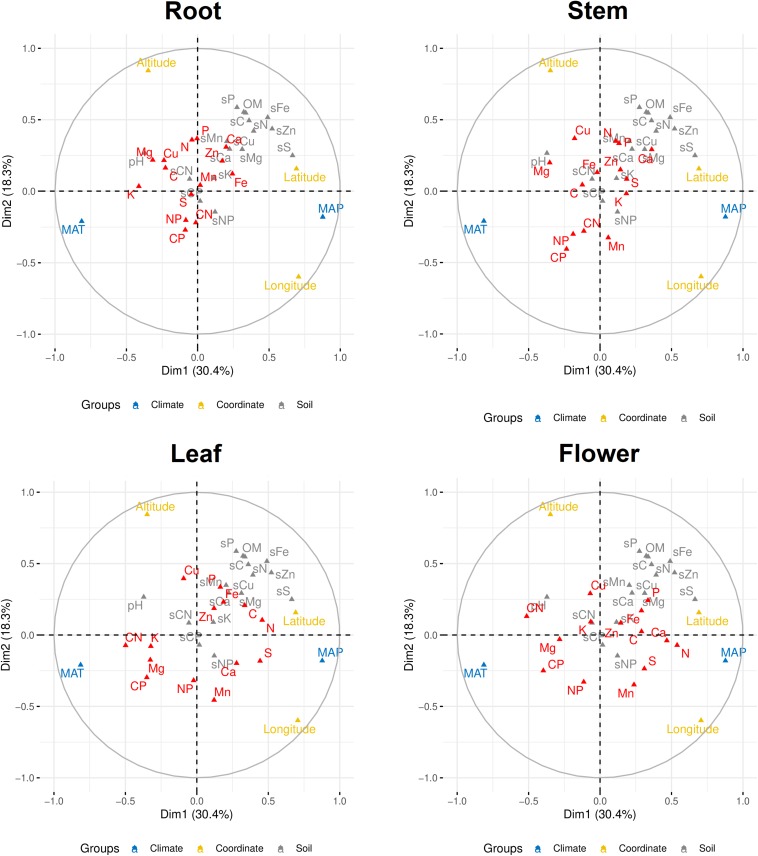
Multiple factor analysis of abiotic environmental factors and multielemental stoichiometry in plant organs. Elements and C:N:P ratios in plants are in red color. MAP, mean annual precipitation; MAT, mean annual temperature; sC, soil carbon; sN, soil nitrogen; sP, soil phosphorus; sK, soil potassium; sCa, soil calcium; sMg, magnesium; sS, soil sulfur; sFe, soil iron; sMn, soil manganese; sCu, soil copper; sZn, soil zinc; sCN, soil C:N ratio; sCP, soil C:P ratio; sNP, soil N:P ratio; OM, soil organic matter, pH, soil pH; CN, plant C:N ratio; CP, plant C:P ratio; NP, plant N:P ratio.

Multiple factor analysis was used to explore the correlations of climate (MAT and MAP), coordinate (latitude, longitude, and altitude), and soil (soil chemical properties) on plant elemental stoichiometry. The first and second dimensions together explained ∼50% of the total variance. The first dimension was mainly contributed by MAP, MAT, latitude, and longitude, while the second dimension was mainly contributed by altitude, longitude, and some soil chemical properties (such as soil P) (Figure 7 and Supplementary Figures S1, S2). Meanwhile, plant stoichiometry responded differently to abiotic environment factors, depending on organ type and element (Figure 7). For example, C:N and C:P ratios were more influenced by MAT in both leaf and flower than those ratios in root, while N content was more influenced by MAP and latitude in both leaf and flower than its content in root. However, N:P ratios in all of the organs seemed not influenced by climate factors associated with geography.

## Discussion

### Inter- and Intraorgan Multielement Stoichiometry

Our data showed that the means of leaf N and P contents of *G. rigescens* are 1.29 and 0.134%, respectively, which are lower than those for global herbaceous plants (2.17 and 0.164%, respectively) and for the herbaceous species (2.09 and 0.155%, respectively) in China (Han et al., 2005; Tian et al., 2018). However, the leaf N:P atomic ratio (26:1) of *G. rigescens* is very similar with 28:1 for N:P ratio in global forests and 30:1 for herb species in China (McGroddy et al., 2004; Han et al., 2005). Garten (1976) found that correlations between contents of elements (such as N and P) across many species were probably because of biochemical similarities of cell metabolism. Moreover, herbaceous plant species tend to maintain their own N and P composition even growing in different sites (Hu et al., 2018). The exponent of leaf N vs. P scaling relationships of global herbaceous plants was 0.659 (Tian et al., 2018). Geng et al. (2014) reported that slopes of N–P scaling did not differ between leaves and fine roots in 139 species collected from Tibetan alpine grassland and Mongolian temperate grassland. In another study, there were no significant differences in the N and P scaling exponents among root, stem, and leaf in 304 species of herbaceous plants (Zhang et al., 2018a). Our data further showed that the slopes of N and P stoichiometric relationships among organs (root, stem, leaf, and flower) of *G. rigescens* did not differ significantly between environments. In *G. rigescens* flower, P and N contents were higher than the values in other plant organs, indicating that flower is the most active organ at the organ level.

We also found that Zn had positive correlations with Fe, S, or Cu in each plant organ. Zn homeostasis is maintained by a tightly regulated network, which includes low molecular-weight ligands, membrane transport and Zn-binding proteins, and so on (Sinclair and Krämer, 2012). Interactions between Zn and Fe homeostasis have been observed in different plant species (Xie et al., 2019). Zn homeostasis interacts with Fe homeostasis as a result of the chemical similarity between their divalent cations and the generality of the key root iron uptake transporter (Sinclair and Krämer, 2012). Zn, Fe, and Cu play roles in plant photosynthesis and the mechanisms that their homeostasis within chloroplasts has been concerned (Yruela, 2013). Moreover, the interaction between Zn and S is a critical biological partnership in which sulfur gives mobility to zinc and zinc adjusts the chemical properties of sulfur (Maret, 2004). Further studies are needed on the interactions of multielement homeostasis in plants.

### Multielemental Stoichiometry Between Plant and Soil

At the global scale, atomic C:N:P ratios in the soil is 186:13:1 on average (Cleveland and Liptzin, 2007). In this study, the soil C:N:P stoichiometry was 169:8:1. It indicated that soils in our study area are more N and P limited, compared with global data. This is in agreement with the finding that soil P contents across most areas of China were below the global average (Han et al., 2005). Strong positive correlations between soil and plant tissue C:N:P stoichiometry were found in field experiment for herbaceous plants (Bell et al., 2014). In a subtropical mountainous region of southwest China, community leaf P was primarily determined by soil P; leaf P increased as soil P availability increased (Yan et al., 2015). In the P-rich soil in a forest in Yunnan, soil P content is a major driver triggering the variation in multielemental stoichiometry in plants (Li et al., 2019). Our study showed that, in the P-limited soil, soil P content also plays an important role in multielemental stoichiometry in plant.

Multielemental stoichiometry in plants can be affected by soil nutrient availability and plant functions that resist to nutrient stress (Tian et al., 2019). Our analyses indicate that the contents of macroelements, such as C, N, P, Ca, Mg, and S, were higher in plant organs than those in soil and exhibited a relatively narrow range in plant organs, which is in agreement with the studies of Han et al. (2011) and Zhao et al. (2016). Both N and P contents in leaf and flower of *G. rigescens* showed stoichiometric relationships with their contents in soil. However, flower and leaf N and P contents of common reed (*Phragmites australis*) did not change significantly with N and P availability in northern China (Li et al., 2014). Further work is needed on the stoichiometry of reproductive tissue in different plant species. In this study, higher Mn content was found in leaf, compared with other organs and soil. The reason for this could be that plants that can release carboxylates to mobilize soil P as a phosphorus-acquisition strategy can also mobilize soil Mn, leading to high leaf Mn content (Lambers et al., 2015). Fe is essential for plants, but excess Fe is toxic to plant because of the formation of damaging reactive oxygen species duo to free intracellular Fe. Therefore, maintaining proper Fe homeostasis is crucial for plants (Jeong and Guerinot, 2009). Plant can reduce Fe uptake when Fe is at high levels (Kroh and Pilon, 2019). Intriguingly, we found that, compared with other studied elements, *G. rigescens* reduced Fe uptake from soil and showed the strictest homeostasis. Furthermore, our average leaf Fe content was nearly 1.3 mg/g (Figure 5), which was more than two times higher than the critical leaf content (>0.5 mg/g, dry weight) for Fe toxicity in non-tolerant crop plants (White and Brown, 2010). The results imply that the soils contain relatively high levels of Fe, but *G. rigescens* can resist to excess Fe.

### Variation of Multielemental Stoichiometry With Climatic Conditions

In this study, MAP and MAT associated with geography were the main drivers for plant stoichiometry. Similar result was found in an investigation on the variation of multielemental stoichiometry in *Quercus variabilis* leaves across China (Sun et al., 2015). Our data showed that plant stoichiometry responded differently to abiotic environmental factors, depending on organ type and element. Contents of leaf N and S were closely correlated with MAP. The same pattern had been found in a study investigating more than 700 wild plant species across China (Zhang et al., 2012). A meta-analysis on multielemental stoichiometry including 1,900 plant species across China showed that global warming might have no effect on leaf Ca and Mg but could decrease leaf K, Fe, Mn, and Zn (Tan et al., 2019). We found that leaf K and Mg had a relatively close relationship with MAT. For N:P ratio, a trend of increasing leaf N:P ratio with decreasing latitude for 753 terrestrial plant species was discovered across China (Han et al., 2005). In another study, leaf N:P ratio increased with increasing MAT along the 400 mm isohyet in north China (Tan et al., 2018). In this study, N:P ratio, no matter in which organ, showed little flexibility to climate factors, which may be because factors intrinsic to each plant species play a more important role in controlling N:P ratios than extrinsic factors at a local scale (Luo et al., 2015). However, at global scale, terrestrial plant N:P ratio decreases with increasing precipitation but increases with warming (Yuan and Chen, 2015).

## Conclusion

In conclusion, our analyses indicate that the slopes of N–P scaling among organs did not differ significantly between environments even in flower, the most active organ with highest N and P level. Zn had strong positive correlations with Fe, S, or Cu in each organ. Fe showed the strictest homeostasis in its root when excessive Fe in soil. In this study, MAP and MAT associated with geography, followed by soil P, were the main divers for plant multielemental stoichiometry, while N:P ratio, no matter in which organ, showed little flexibility to climate factors. The results have implications for understanding the roles that multielemental stoichiometry play for plant individuals to nutrient stress and climate change.

## Data Availability Statement

All datasets generated for this study are included in the article/Supplementary Material.

## Author Contributions

JZ, YW, and CC conceived and designed the study. JZ and YW collected the samples. JZ analyzed the samples and analyzed the data. JZ and CC wrote the manuscript.

## Conflict of Interest

The authors declare that the research was conducted in the absence of any commercial or financial relationships that could be construed as a potential conflict of interest.

## Supplementary Material

TABLE S1 Information of the sampling sites.

FIGURE S1 The contribution of quantitative variables (in%) to the first dimension of the multiple factor analysis.

FIGURE S2 The contribution of quantitative variables (in%) to the second dimension of the multiple factor analysis.

DATA SHEET S1 The dataset for this study.
