# Supplementary material for: Multielemental Stoichiometry in Plant Organs: A Case Study With the Alpine Herb Gentiana rigescens Across Southwest China
Source: Front Plant Sci. 2020 Apr 28;11:441. doi: 10.3389/fpls.2020.00441 (PMC7198822; doi:10.3389/fpls.2020.00441)
Supplement: TABLE S1 — Information of the sampling sites. [file Presentation_1.pdf]

# *Supplementary Material*

**Table S1. Information of the sampling sites.**

| Population code | Elevation | Latitude | Longitude | Site |
|---|---|---|---|---|
| 1 | 2978 | N26°34'36.6" | E99°26'32.5" | Nujiang, Yunnan |
| 2 | 2356 | N25°29'33.7" | E99°38'17.5" | Dali, Yunnan |
| 3 | 2852 | N25°51'14.1" | E99°51'20.4" | Nujiang, Yunnan |
| 4 | 2821 | N24°48'39.5" | E99°47'10.2" | Baoshan, Yunnan |
| 5 | 1914 | N24°57'01.1" | E100°28'10.7" | Dali, Yunnan |
| 6 | 2520 | N24°29'40.2" | E100°47'05.8" | Pu'er, Yunnan |
| 7 | 2311 | N23°55'25.5" | E101°06'14.3" | Pu'er, Yunnan |
| 8 | 2302 | N23°56'21.0" | E101°29'05.6" | Yuxi, Yunnan |
| 9 | 2016 | N23°58'01.1" | E101°56'57.1" | Yuxi, Yunnan |
| 10 | 1817 | N25°02'57.7" | E103°17'39.6" | Kunming, Yunnan |
| 11 | 2144 | N24°42'47.6" | E103°36'19.5" | Kunming, Yunnan |
| 12 | 2072 | N25°42'22.1" | E104°10'27.5" | Qujing, Yunnan |
| 13 | 2292 | N25°19'62.57" | E102°52'38.01" | Kunming, Yunnan |
| 14 | 1879 | N24°23'31.34" | E102°40'37.45" | Yuxi, Yunnan |
| 15 | 2318 | N24°30'53.9" | E103°49'42.2" | Honghe, Yunnan |
| 16 | 1891 | N24°24'51.4" | E103°41'16.3" | Honghe, Yunnan |
| 17 | 2015 | N24°15'16.1" | E103°37'26.0" | Honghe, Yunnan |
| 18 | 1993 | N24°02'38.8" | E103°44'26.3" | Wenshan, Yunnan |
| 19 | 1788 | N23°18'58.7" | E104°08'32.3" | Wenshan, Yunnan |
| 20 | 1987 | N23°24'27.4" | E103°43'50.2" | Honghe, Yunnan |

| | | | | |
|---|---|---|---|---|
| 21 | 1945 | N23°46'43.2" | E102°48'49.4" | Honghe, Yunnan |
| 22 | 2205 | N25°23'24.3" | E102°26'47.0" | Kunming, Yunnan |
| 23 | 1804 | N25°40'06.3" | E101°36'44.4" | Chuxiong, Yunnan |
| 24 | 1969 | N25°05'05.4" | E101°16'51.9" | Chuxiong, Yunnan |
| 25 | 1945 | N24°08'19.4" | E102°15'26.1" | Yuxi, Yunnan |
| 26 | 2061 | N25°27'30.0" | E103°36'21.4" | Qujing, Yunnan |
| 27 | 2258 | N27°42'36.8" | E102°21'30.4" | Liangshan, Sichuan |
| 28 | 2601 | N27°51'03.5" | E102°25'50.3" | Liangshan, Sichuan |
| 29 | 1921 | N28°31'27.2" | E102°12'19.3" | Liangshan, Sichuan |
| 30 | 2807 | N27°09'35.0" | E101°16'26.5" | Panzhihua, Sichuan |
| 31 | 1663 | N27°11'51.3" | E105°42'23.0" | Bijie, Guizhou |
| 32 | 1454 | N26°56'00.0" | E105°55'54.0" | Bijie, Guizhou |
| 33 | 1895 | N26°43'06.1" | E105°16'40.5" | Bijie, Guizhou |
| 34 | 2473 | N26°38'29.4" | E104°42'37.5" | Liupanshui, Guizhou |
| 35 | 1990 | N27°04'59.9" | E104°38'05.0" | Bijie, Guizhou |
| 36 | 2018 | N27°18'21.2" | E104°45'57.3" | Bijie, Guizhou |
| 37 | 1590 | N26°38'22.6" | E104°43'26.0" | Guiyang, Guizhou |
| 38 | 1598 | N25°07'00.7" | E104°43'38.2" | Qianxinan, Guizhou |
| 39 | 1800 | N25°22'58.0" | E105°07'19.5" | Qianxinan, Guizhou |
| 40 | 1393 | N26°33'33.6" | E106°54'59.2" | Qiannan, Guizhou |
| 41 | 1300 | N26°27'38.7" | E106°55'40.8" | Qiannan, Guizhou |
| 42 | 1231 | N26°23'58.7" | E107°58'18.3" | Qiandongnan, Guizhou |
| 43 | 1577 | N26°22'43.7" | E108°11'08.0" | Qiandongnan, Guizhou |
| 44 | 1260 | N26°33'36.2" | E108°19'45.5" | Qiandongnan, Guizhou |
| 45 | 2036 | N24°58'11" | E104°45'46" | Xingyi, Guizhou |

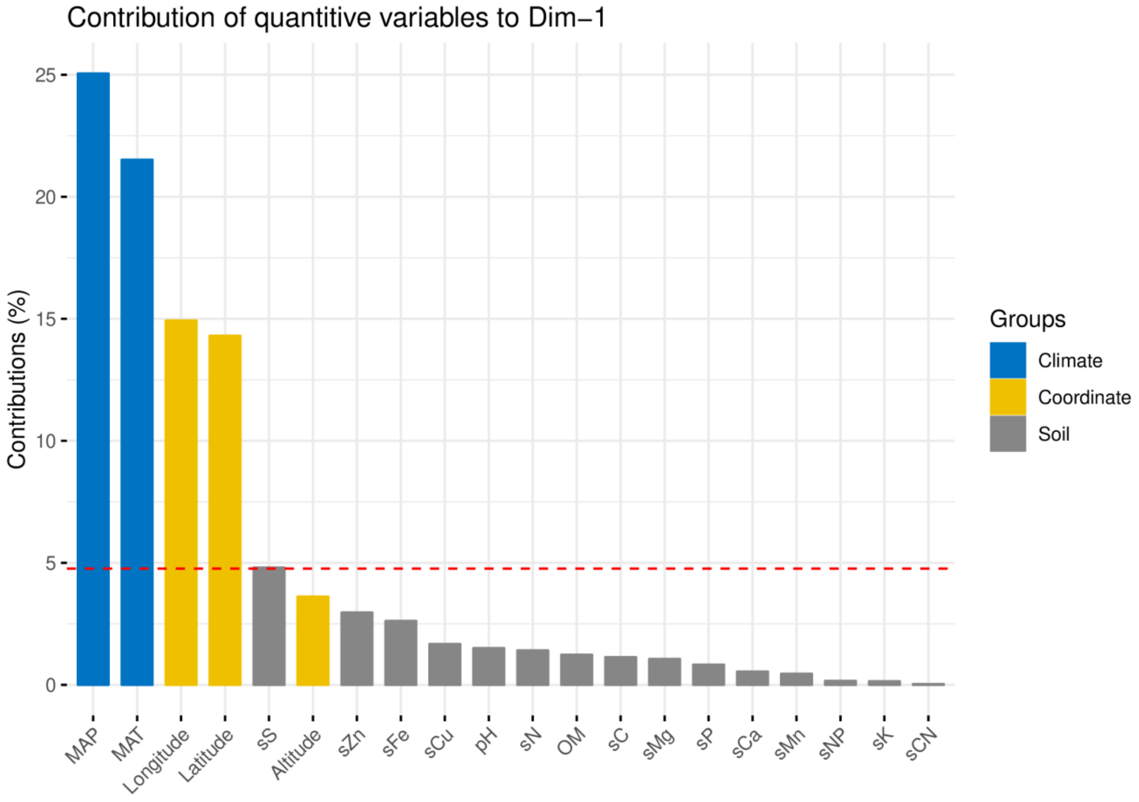


**Figure S1. The contribution of quantitative variables (in %) to the first dimension of the multiple factor analysis.**

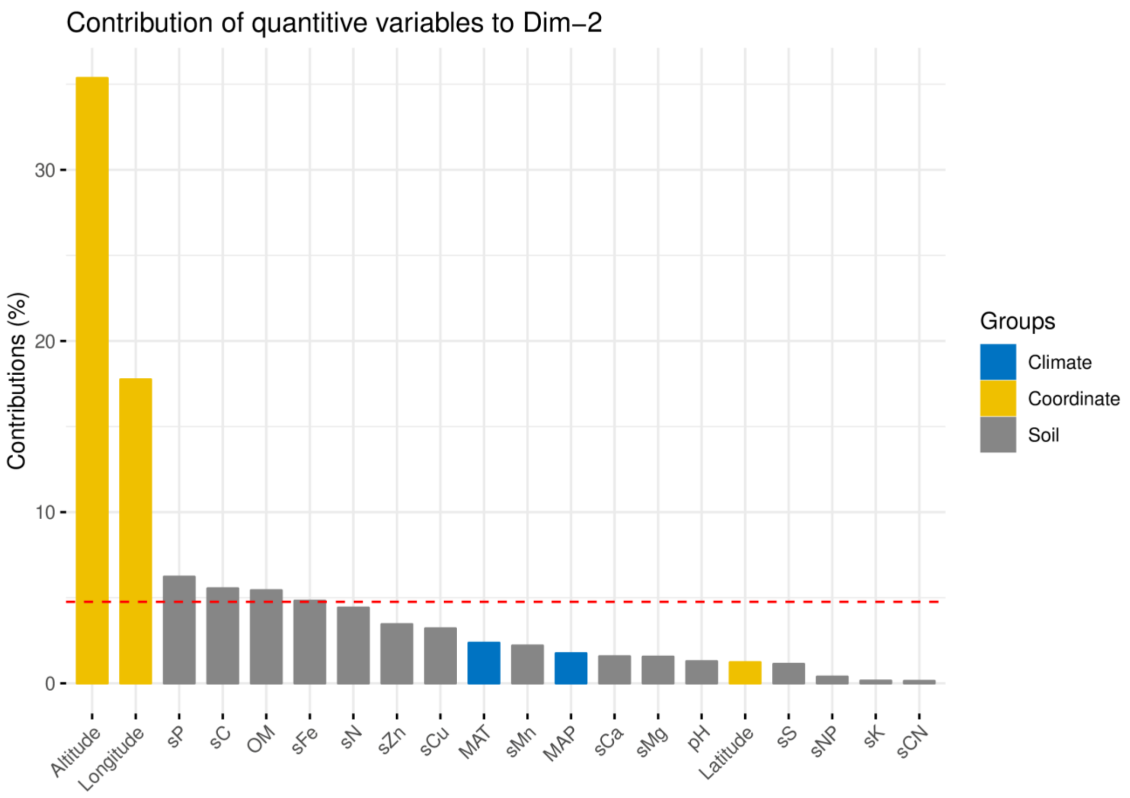


**Figure S2. The contribution of quantitative variables (in %) to the second dimension of the multiple factor analysis.**
